# Seasonal dynamics of lotic bacterial communities assessed by 16S rRNA gene amplicon deep sequencing

**DOI:** 10.1038/s41598-020-73293-9

**Published:** 2020-10-02

**Authors:** Lisa Paruch, Adam M. Paruch, Hans Geir Eiken, Monica Skogen, Roald Sørheim

**Affiliations:** 1 Division of Environment and Natural Resources, Norwegian Institute of Bioeconomy Research – NIBIO, Oluf Thesens vei 43, 1433 Ås, Norway; 2 Division of Biotechnology and Plant Health, Norwegian Institute of Bioeconomy Research – NIBIO, Høgskoleveien 7, 1433 Ås, Norway

**Keywords:** Sequencing, DNA sequencing, Next-generation sequencing, Environmental biotechnology, Microbial ecology, Microbiome, Applied microbiology, Bacteria, Microbial communities, Environmental microbiology, Biotechnology, Microbiology, Biodiversity, Freshwater ecology, Microbial ecology, Ecology, Population dynamics

## Abstract

Aquatic microbial diversity, composition, and dynamics play vital roles in sustaining water ecosystem functionality. Yet, there is still limited knowledge on bacterial seasonal dynamics in lotic environments. This study explores a temporal pattern of bacterial community structures in lotic freshwater over a 2-year period. The aquatic bacterial communities were assessed using Illumina MiSeq sequencing of 16S rRNA genes. Overall, the communities were dominated by α-, β-, and γ-*Proteobacteria, Bacteroidetes*, *Flavobacteriia*, and *Sphingobacteriia*. The bacterial compositions varied substantially in response to seasonal changes (cold vs. warm), but they were rather stable within the same season. Furthermore, higher diversity was observed in cold seasons compared to warm periods. The combined seasonal-environmental impact of different physico-chemical parameters was assessed statistically, and temperature, suspended solids, and nitrogen were determined to be the primary abiotic factors shaping the temporal bacterial assemblages. This study enriches particular knowledge on the seasonal succession of the lotic freshwater bacteria.

## Introduction

Aquatic bacteria play an essential and vital role in sustaining natural water ecosystems through a variety of activities involved in fundamental biogeochemical processes, such as organic and inorganic nutrients’ cycling, respiration, and attenuation of biological and chemical pollutants in aquatic environments^1^. However, under certain circumstances/extreme stress, these bacteria could, in turn, have an adverse impact on the ecosystem by causing water quality deterioration, for example, or in worse cases, creating serious health problems (waterborne pathogens) for both humans and animals^2–5^. Apparently, aquatic microbiota associate most closely with biological water quality and public and environmental health. Therefore, a comprehensive understanding of aquatic microbial diversity, composition, and dynamics are of essential importance for water quality assessments, predictions, and management strategies for sustainable ecosystem functions^6,7^.

A number of published data have illustrated spatial heterogeneity of bacterial communities in freshwater ecosystems^8–10^. Bacterial population’s seasonality in different water types and variant ecosystems, such as bacterioplankton communities^11,12^, bacterial communities in sediments^13^, wastewater treatment plants^14,15^, and drinking water works^16,17^, as well as in the estuarine ecosystems^18,19^, has repeatedly been observed. However, seasonal impacts on the lotic bacterial community structure remain sparsely addressed.

To investigate the aquatic microbial community structure and dynamics, various molecular approaches have been adopted to address specific targets of interest. For instance, polymerase chain reaction-denaturing gradient gel electrophoresis (PCR-DGGE) and 16S rRNA deep sequencing have been applied to study bacterial communities in tropical freshwaters^20^. Also, an automated ribosomal intergenic spacer analysis (ARISA), as a DNA fingerprinting methodology, was utilized to investigate bacterial communities in lentic freshwater^8^. More recently, with the advancement of next generation sequencing (NGS) technologies, unprecedented high-throughput and in-depth data resolution enable faster and better interpretation of the microbial makeup in different types of water. Illumina sequencing platforms (MiSeq and HiSeq) have become favourable tools. For instance, MiSeq was applied to study the shift of the microbial community composition in an urban river and detected spatial variation in both surface water and sediment^9^. Spatiotemporal changes of a bacterial community and the microbial activity of a full-scale drinking water treatment plant were determined by employing Illumina HiSeq sequencing, together with cultivation-based methods^17^. Evidently, by providing extremely high and in-depth data throughput, Illumina sequencing-based analyses could greatly facilitate various microbial ecology studies.

Since the seasonal patterns of bacterial diversity in lotic ecosystems are marginally reported, we set up field and laboratory research to study the temporal dynamics of freshwater bacteria in a Norwegian rural creek using Illumina MiSeq sequencing of 16S rRNA genes. The core research questions we set out to address were the following: (i) do lotic microbial populations in the creek exhibit the seasonality? If yes, (ii) is there any dynamic pattern followed during the main seasonal shift (cold vs. warm)? (iii) which abiotic environmental factors contribute significantly to shaping the microbial community structures in the studied lotic water? Outcomes of the addressed study could fill in the knowledge gap regarding the combined temporal-environmental effect on lotic bacterial community structures.

## Results and discussion

### Aquatic bacterial community analysis

**Figure 1:**
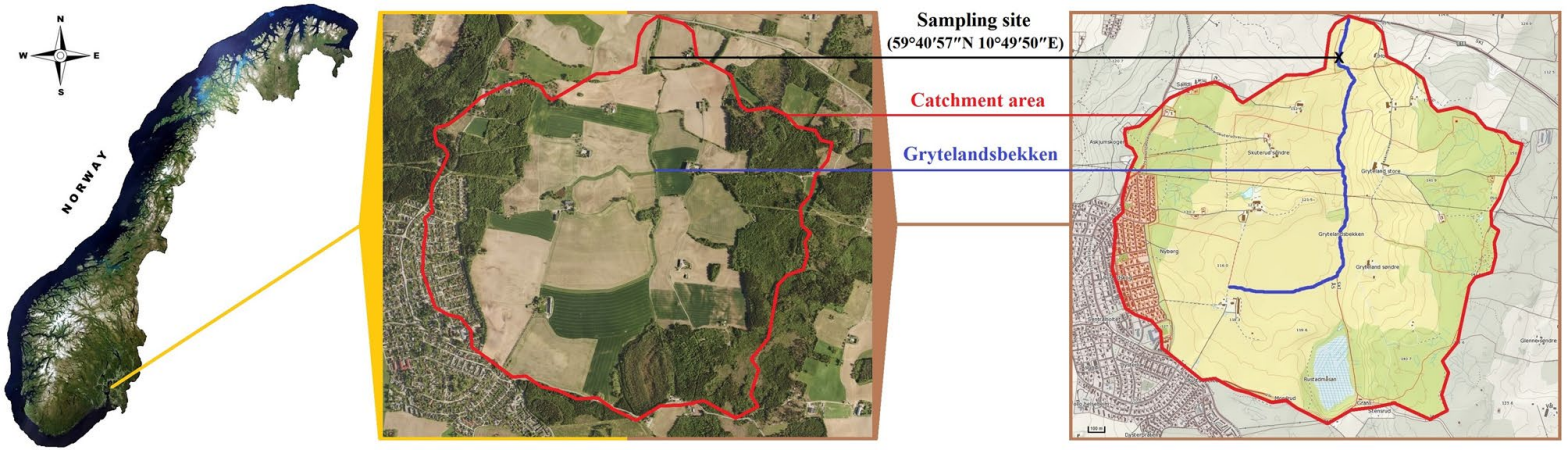
Location of the study site, Grytelandsbekken rural creek in the municipality of Ås. The satellite image and map were obtained from NIBIO’s primary map service, the Source/Kilden (https://kilden.nibio.no/?lang=nb&topic=arealinformasjon&bgLayer=norgeibilder_cache2&X=7334000.00&Y=400000.00&zoom=0).

The bacterial community compositions were investigated in a Norwegian rural creek named Grytelandsbekken (Fig. 1), which was previously characterised as a lotic ecosystem with relatively low levels of anthropogenic faecal pollution and a very high microbial diversity and richness profile^21^. The amplicon libraries were prepared on the extracted genomic DNA from the lotic water samples. For sequence data processing, 4.7 million reads were left after trimming and quality filtering, and on average, each sample obtained 98,000 “clean” reads. A total of 160,674 unique reads were used to classify 13,322 operational taxonomic units (OTUs). Based on the characterised OTUs, 65 bacterial phyla, 204 classes, 406 orders, 644 families, and 1,068 genera were classified. Prevalently high proportions (80–90%) of bacterial populations were assigned to *Proteobacteria* and *Bacteroidetes* phyla across all examined samples, despite seasonal changes. Among them, α-, β-, and γ-*Proteobacteria* were dominant classes of *Proteobacteria* phylum, while *Flavobacteriia* and *Sphingobacteriia* were prevailing in the *Bacteroidetes* phylum. Similar findings were recently reported by Azmuda et al.^20^, where these two phyla were among the major bacterial groups of lentic freshwater ecosystems and did not reveal seasonal variations. However, these findings somewhat deviated from discoveries of Lee et al.^22^ in marine ecosystems, where *Proteobacteria and Bacteroidetes* only dominated from June to November, while in December, the dominance switched to *Firmicutes*. This discloses that in freshwater environments, whether lotic (flowing waters, as exemplified in our study case) or lentic (standing waters, e.g. ponds and lakes), the bacterial temporal dynamic is more stable than in marine environments.

### Seasonal succession of bacterial community

**Figure 2:**
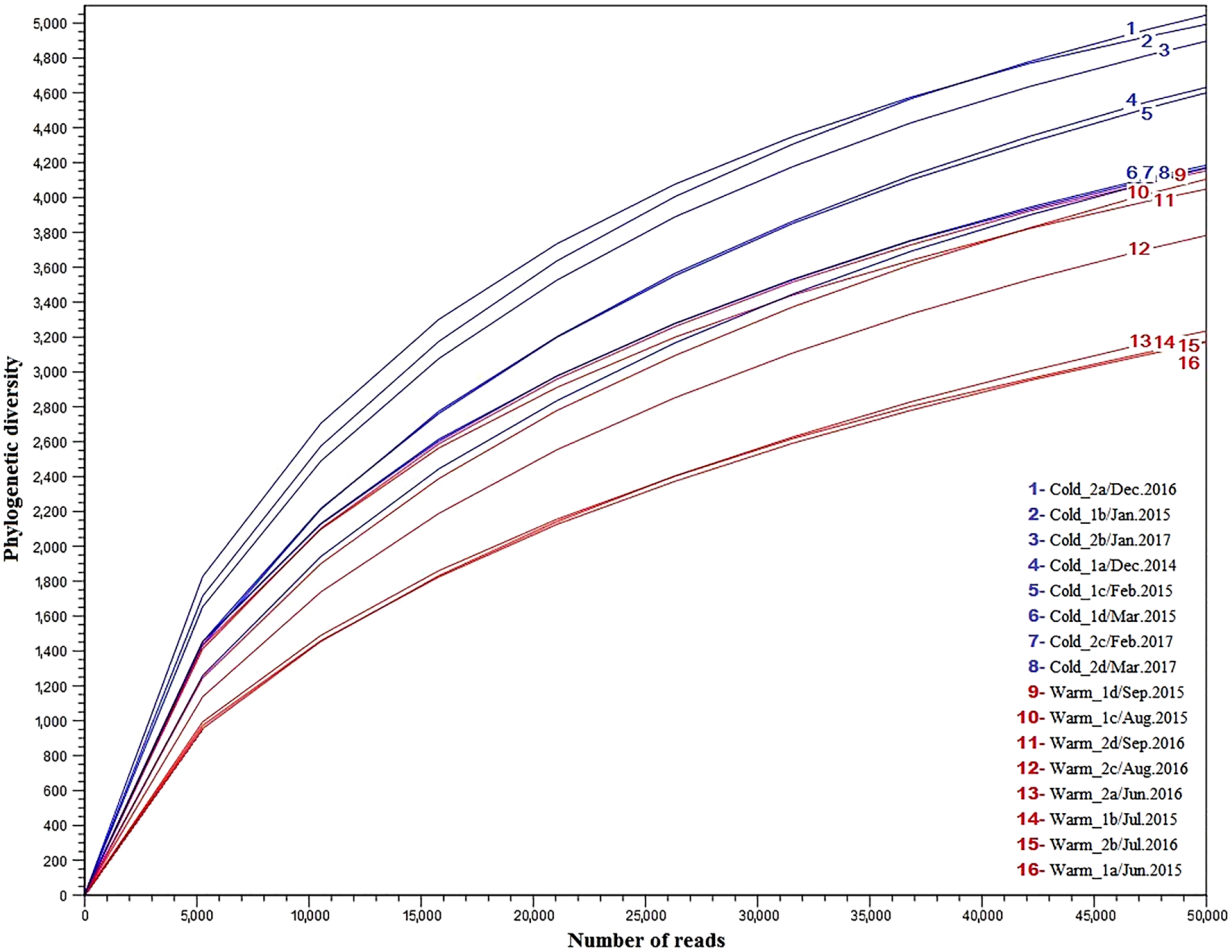
Phylogenetic alpha diversity of the bacterial population through all the cold-warm seasonal regimes. The alpha rarefaction plot was generated using the tools in CLC Microbial Genomics Module version 2.5.1 (CLC Bio, QIAGEN Company, Aarhus, Denmark, https://www.qiagenbioinformatics.com/clc-microbial-genomicsmodule-latest-improvements).

The alpha diversity measured by rarefaction analysis (Fig. 2) revealed a distinct seasonal pattern of bacterial population dynamics. In general, the bacterial diversities varied from cold to warm seasons. Moreover, higher richness and diversity were detected in cold than in warm periods, and these were statistically significant (Table 1). This trend was observed across all sampling events over the course of the 2-year study (see Supplementary Data). The same tendency was also noticed by Kaevska et al.^23^ in a river ecosystem and Suh et al.^24^ in a marine bay. One of the factors favouring this trend is related to the long and strong solar UV radiation during the summer season, which leads to reduced microbial diversity^25,26^.

**Table 1:** Mean ± standard deviation of alpha diversity indices in the cold seasons (Dec.2014–Mar.2015 and Dec.2016–Mar.2017) and warm seasons (Jun.2015–Sep.2015 and Jun.2016–Sep.2016).

Indices	Cold	Warm	p-value
Chao 1	7271 ± 556	5929 ± 707	0.001
Shannon entropy	9.25 ± 0.67	8.3 ± 0.72	0.016
Richness (total OTUs)	5185 ± 552	3955 ± 539	0
Phylogenetic diversity	8.51 ± 0.38	7.36 ± 0.74	0.002
Inverse Simpson Index	1.02 ± 0.008	1.02 ± 0.007	0.764

The indices were generated in CLC Microbial Genomics Module version 2.5.1 (CLC Bio, QIAGEN Company, Aarhus, Denmark, https://www.qiagenbioinformatics.com/clc-microbial-genomicsmodule-latest-improvements) and statistically tested using a two-tailed Student’s t-test at 0.05 significance in the XLSTAT-ECOLOGY statistical software package version 2019.1.1 (Addinsoft 2020, Boston, USA, https://www.xlstat.com).

**Figure 3:**
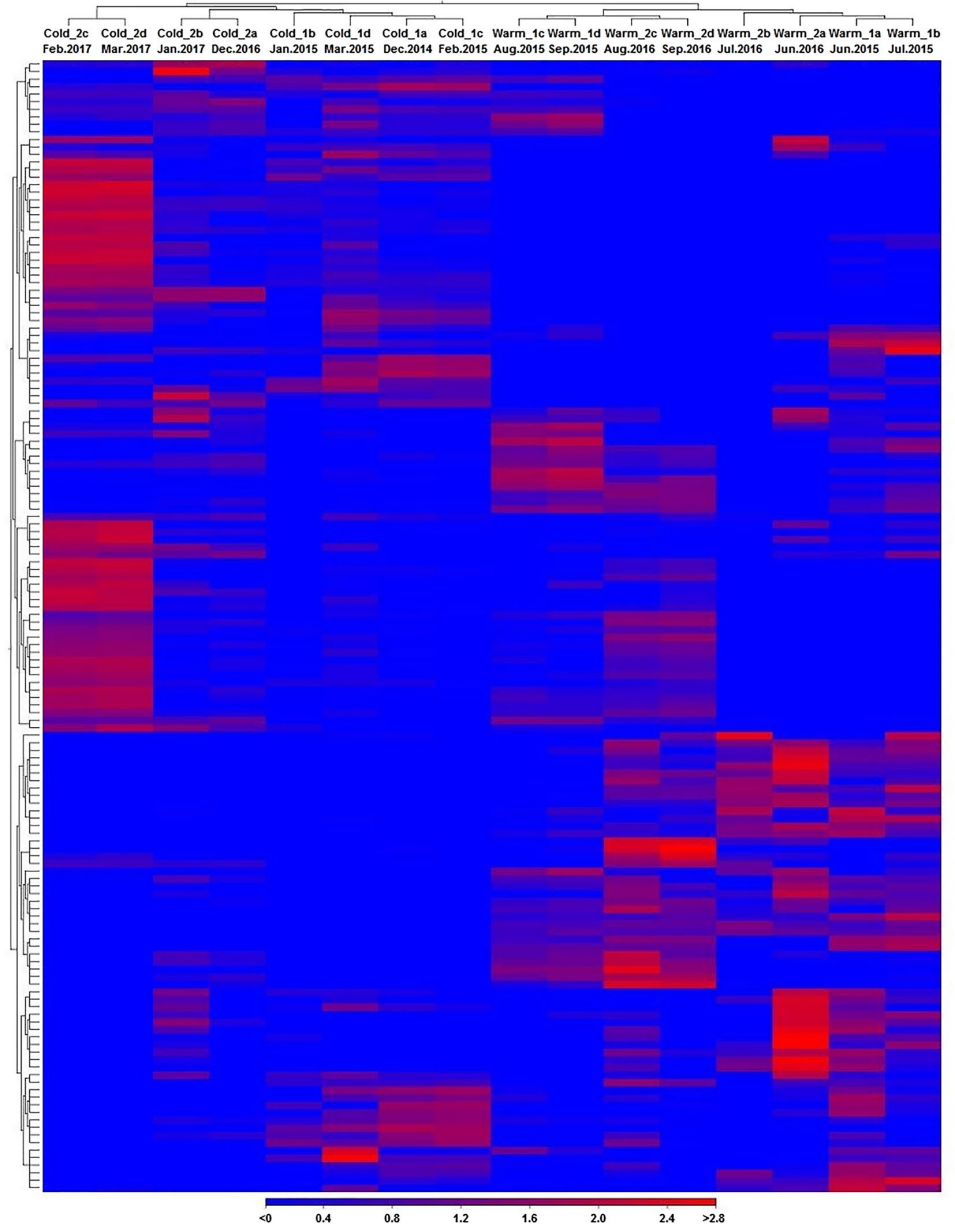
Beta diversity heat map with hierarchical clustering of bacterial communities through all the cold-warm seasonal regimes. The hierarchical clustering heat map was generated using the tools in CLC Microbial Genomics Module version 2.5.1 (CLC Bio, QIAGEN Company, Aarhus, Denmark, https://www.qiagenbioinformatics.com/clc-microbial-genomics-module-latest-improvements).

**Figure 4:**
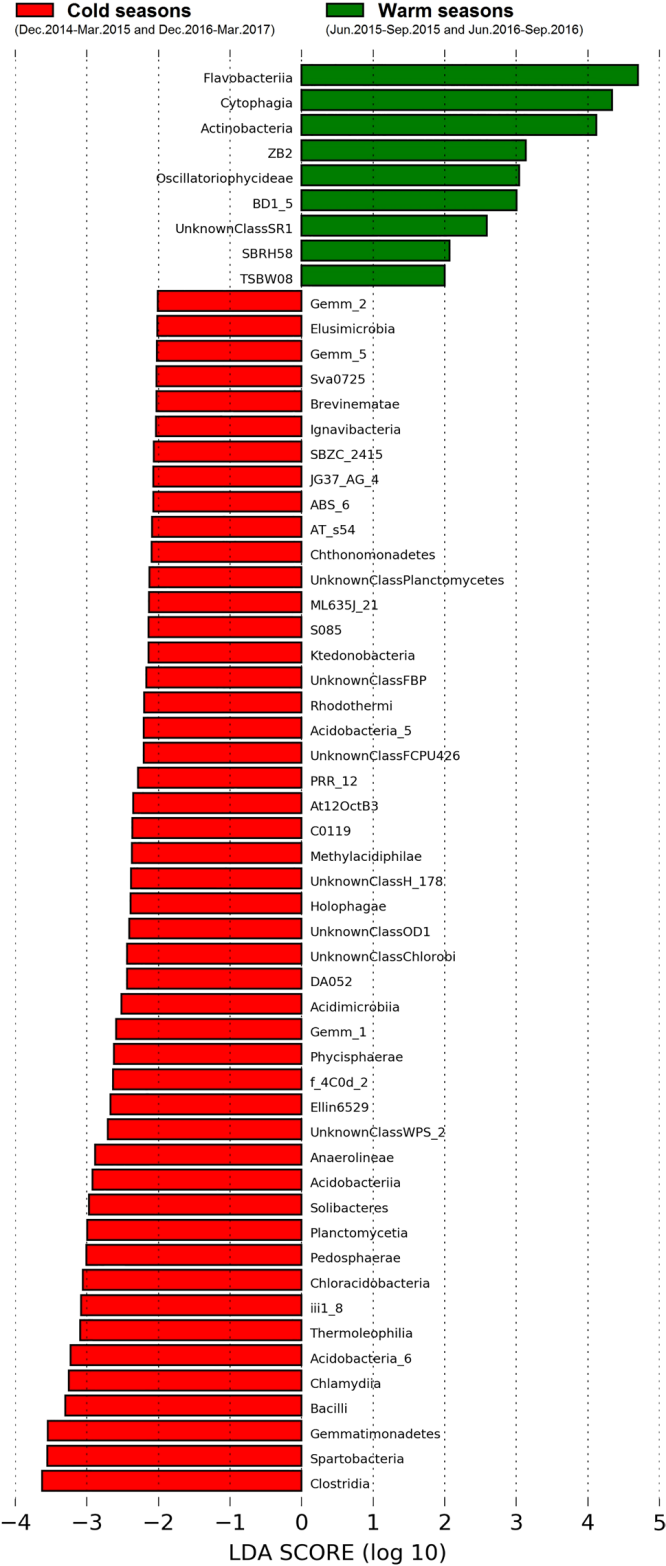
LEfSe rank plot of differentially abundant bacterial classes in the cold and warm seasons, expressed as LDA logarithmic scores. The LEfSe plot was generated using the tools in Galaxy version 1.0 (the Huttenhower Lab, Harvard School of Public Health, Boston, MA, USA, https://huttenhower.sph.harvard.edu/galaxy).

The beta diversity heat map analysis of the top 700 most abundant OTUs (determined based on ranked relative abundances) revealed a distinctive seasonal classification (Fig. 3). All cold seasons’ samples were closely related in community signatures and clustered together. The other separate cluster was comprised of all warm seasons’ samples. PERMANOVA analysis demonstrated that these clusters were statistically significant (p-value = 0.00001). This distinct clustering pattern exhibited a determined temporal dynamic of bacterial communities in the studied lotic ecosystem. Yet, some taxa were common to cold and warm seasons (see Supplementary Fig. S1). Most of them are affiliated to *Proteobacteria*, which are ubiquitous, persistent and prevalent in freshwater ecosystems under various environmental conditions^16,20,21^. To focus in on the population discrepancy of the bacterial community between cold and warm seasons, a linear discriminant analysis (LDA) effect size (LEfSe) tool was performed on the bacterial class level for the two aligned groups (cold vs. warm), generated by merging all taxa from the same seasonal samples (Fig. 4). The assay unravelled the principle bacterial members responsible for the significant community structural discrepancy following the seasonal shift (the Wilcoxon test) and discovered more diverse bacteria distributed during cold seasons than in warm periods. *Clostridia* and *Bacilli*, affiliated to *Firmicutes* phylum in cold periods and *Flavobacteriia* and *Cytophagia* classes of *Bacteroidetes* in warm seasons, had the highest LDA scores and represented the leading bacterial members driving the seasonal succession. These findings were in line with a recent report by Lee et al.^22^ on temporal dominancy of *Firmicutes* and *Bacteroidetes* (although in marine waters) during cold and warm periods, respectively.

### Bacterial structure in response to environmental factors

**Figure 5:**
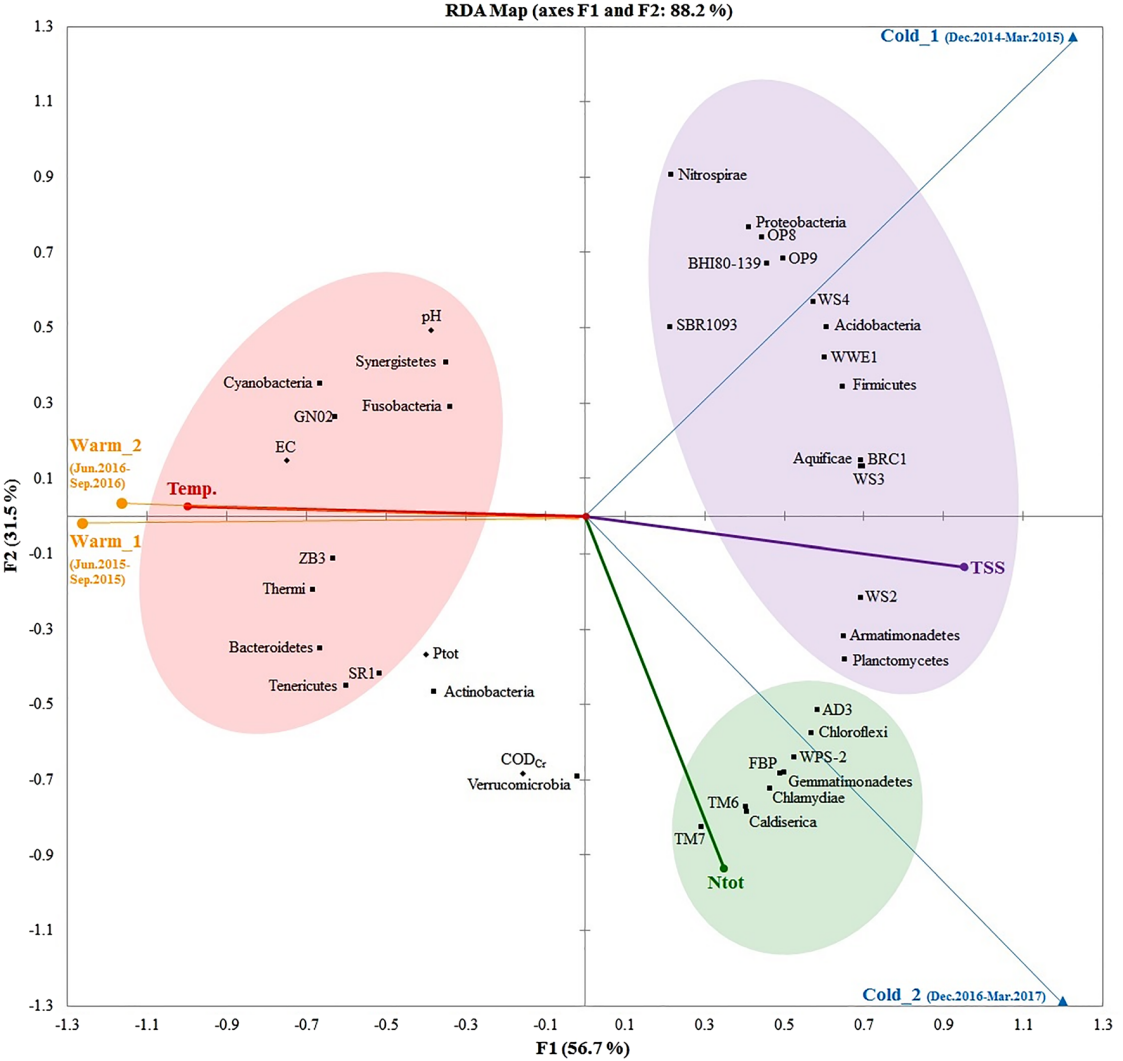
RDA plot incorporated with clusters of significant (p < 0.05) relationships between the lotic bacterial communities, seasonal changes (cold and warm), and environmental variables (temperature—Temp, total suspended solids—TSS, and total nitrogen—Ntot). The RDA plot was generated using the tools in the XLSTAT-ECOLOGY statistical software package version 2019.1.1 (Addinsoft 2020, Boston, USA, https://www.xlstat.com).

Redundancy analysis (RDA) explored the correlation between examined biotic and abiotic components in relation to temporal patterns. The resulting RDA map (Fig. 5) explained the high, over 88%, variability detected. Correlation scores per parameters indicated that temperature (Temp), total suspended solids (TSS), and total nitrogen (Ntot) had the most significant impacts (p < 0.05 in Pearson correlation test) on the bacterial diversity. Some phyla, such as *Thermi*, *Cyanobacteria*, *Bacteroidetes*, *Tenericutes*, *ZB3,* and *GN02*, were evidently more prevalent in warm seasons, while quite a large number of diverse bacteria revealed negative relationships with temperature. More interestingly, the majority of bacteria were identified in cold periods and positively correlated with TSS (Fig. 5). This illustrates that Temp and TSS exhibited strong opposite impacts on the seasonality of the bacterial diversity in the studied lotic water. Furthermore, members of phyla, such as *Caldiserica*, *Chlamydiae*, *Chloroflexi*, *FBP*, *Gemmatimonadetes*, *TM6,* and *TM7* (known as *Saccharibacteria*), responded positively to the Ntot level and were more abundant in cold seasons. Other environmental factors, such as chemical oxygen demand (COD_Cr_), electrical conductivity (EC), and pH (Table 2), were less relevant through the entire course of study; though, this effect of pH was largely due to the low fluctuations/relatively stable measurements (between neutral and mildly basic pH, Table 2). It is worth noting that these key environmental variables contributed profoundly to shaping the bacterial community of the studied lotic freshwater under definite temporal impacts.

**Table 2:** Physico-chemical characteristic of the lotic freshwater environment at the study site, Grytelandsbekken rural creek in the municipality of Ås.

Environmental factors	Values of the factors through all sampling events															
	Cold_1a Dec.2014	Cold_1b Jan.2015	Cold_1c Feb.2015	Cold_1d Mar.2015	Warm_1a Jun.2015	Warm_1b Jul.2015	Warm_1c Aug.2015	Warm_1d Sep.2015	Warm_2a Jun.2016	Warm_2b Jul.2016	Warm_2c Aug.2016	Warm_2d Sep.2016	Cold_2a Dec.2016	Cold_2b Jan.2017	Cold_2c Feb.2017	Cold_2d Mar.2017
Temperature (Temp)	2.6	1.4	4.1	4.2	13.7	18.7	19.6	11.5	20.4	17.8	18.9	12.4	0.7	0.9	3.9	2.1
Total suspended solids (TSS)	18.4	37.6	5	6.7	5.6	6.2	9.6	10	5.7	12.7	5.7	6.6	7.2	9.4	17.6	16.2
Total nitrogen (TN)	3.47	3.48	3.06	3.87	3.37	3.28	4.48	4.42	3.98	2.93	3.5	3.91	7.48	6.65	4.02	3.83
Total phosphorus (TP)	0.059	0.065	0.03	0.033	0.228	0.049	0.056	0.061	0.031	0.036	0.06	0.065	0.048	0.027	0.12	0.123
Chemical oxygen demand (COD_cr_)	19	27	15	11	22	20	38	40	9	12	27	29	27	26	29	27
Electrical conductivity (EC)	16.5	16.3	18.3	18.5	24	28	16	16.5	31.6	31.6	28.1	28	16.6	16.7	14	14.2
pH	7.35	7.39	7.53	7.5	7.43	7.4	7.1	7.09	7.67	7.47	7.7	7.45	7.35	7.39	7.07	7.01

Values of environmental factors in °C, mg/l and mS/m.

Temperature has often been identified as one of the major abiotic environmental variables contributing, significantly, to shaping aquatic microbial communities. For instance, Mai et al.^12^ identified that temperature is responsible for the shifts of bacterial community in the Pearl River estuary. Similarly, temperature drove spatiotemporal variation of the bacterial composition in freshwater Lake Taihu^27^. In response to changes of nutrient levels in water, both Ntot and total phosphorus (Ptot) have frequently been reported together as the two principle nutrient impact factors responsible for the seasonality of the aquatic bacterial community^24,28,29^. However, we found that in the studied lotic water, Ntot imposed a stronger impact than Ptot on bacterial diversity. Though, *Actinobacteria* demonstrated a remarkably positive correlation with Ptot (Fig. 5), which could very well be linked to their active involvement in phosphate solubilization^30,31^. Notably, the major seasonal dynamic of bacterial assemblage was driven, significantly, by TSS (Fig. 5). This is in concordance with reports from recent studies reporting TSS as the primary environmental abiotic driver shaping the diversity and composition of bacteria in freshwaters of different climatic zones^32–34^. Both lotic bacterioplankton and lentic bacterial communities in subtropical waters were considerably structured under the impact of TSS^33,34^. It was also identified as one of the crucial environmental factors affecting polar bacterial composition in a combined lentic-lotic ecosystem, an arctic lake-stream system^32^.

Overall, there is, by far, very limited published data solely addressing the bacterial population dynamics in lotic environments. Therefore, the results of our study are coherently argued and aligned to only some relevant scientific outputs on temporal patterns of aquatic bacteria, though in various water (fresh and marine) ecosystems. We discovered distinct seasonal changes in bacterial communities of the studied lotic ecosystem, indicating much higher diversity and abundance in cold than warm periods; though, the communities were rather stable during the same season. Furthermore, our study revealed that the temporal bacterial assemblages were strongly shaped by the impact of a few abiotic environmental factors, mostly suspended solids, temperature, and nitrogen. To conclude, our research presents a novel interpretation of the seasonal heterogeneity of the bacterial community and the associated environmental factors, validated by both analytical and statistical analyses. The outcomes provide deeper and novel insights into the seasonal dynamics of bacterial communities in lotic freshwater ecosystems.

## Materials and methods

### Sampling site and regimes

Field work was carried out in the agricultural catchment of Grytelandsbekken (a creek of approx. 2.5 km in length) also known as Skuterud catchment, located in the municipality of Ås (20,000 people), 30 km southeast of Oslo (Fig. 1). The catchment area is of approx. 4.5 km^2^ and largely consists of farmlands (60%) and forest/marshlands (31%). Grytelandsbekken has previously been studied for spatial variations in the microbial communities of various lotic freshwater ecosystems in different regions of Norway^21^. Based on that study, it was characterised as a rural creek with the highest diversity and abundance of microbial communities. Thus, in this follow-up study, Grytelandsbekken, specifically, was selected for assessing the seasonal dynamics of lotic freshwater bacterial communities.

All field measurements and samplings were carried out over a 2-year period at the same site of the creek, i.e. at about 0.25 km. In total, there were 16 sampling events, split equally between cold and warm seasonal regimes. The cold season refers to December–March, while the warm season includes June–September. In detail, there were four events during the cold season in the first year, Cold_1 (Cold_1a/Dec.2014, Cold_1b/Jan.2015, Cold_1c/Feb.2015, and Cold_1d/Mar.2015) and four events during the cold season in the second year, Cold_2 (Cold_2a/Dec.2016, Cold_2b/Jan.2017, Cold_2c/Feb.2017, and Cold_2d/Mar.2017). A similar sample assembly was applied to the warm seasonal regimes, i.e. four in the first year, Warm_1 (Warm_1a/Jun.2015, Warm_1b/Jul.2015, Warm_1c/Aug.2015, and Warm_1d/Sep.2015) and four in the second year, Warm_2 (Warm_2a/Jun.2016, Warm_2b/Jul.2016, Warm_2c/Aug.2016, and Warm_2d/Sep.2016). The entire study duration and sampling sets were conceived based on similar settings reported in aquatic research worldwide, profiling microbial communities through seasonal and spatial variations^20,35,36^.

### Environmental measures

Primary physico-chemical parameters that are routinely measured in standard catchment water quality control^37,38^ were selected for abiotic characteristics of the lotic environment. These included organic matter content (expressed as COD_Cr_), TSS, nutrients (Ptot and Ntot), EC, pH, and Temp. The latter was measured in situ at one of the weather stations, administrated by the Norwegian Institute of Bioeconomy Research (NIBIO) and located at the field measuring/sampling site of Grytelandsbekken. The station is equipped with a number of automatic sensors, providing hourly measurements and registering various climatic data online, which are all open and available at the AgroMetBase hosted by NIBIO (https://lmt.nibio.no/agrometbase/getweatherdata.php). The former parameters were analysed post-sampling in an accredited laboratory of the ALS Laboratory Group Norway AS. These analyses were performed in accordance with ISO and national standards for the respective parameters: COD_Cr_ (ISO 15705), TSS (CSN EN 872, NS 4733), Ptot (ISO 6878, ISO 15681-1), Ntot (EN 12260), EC (EN 27 888, SM 2520B, EN 16192), and pH (ISO 10523, EPA 150.1, EN 16192).

### Genomic DNA purification: 16S rRNA amplicon library preparation and MiSeq sequencing

The seasonal water samples underwent DNA extraction using the QIAGEN DNeasy PowerWater Kit (QIAGEN GmbH, Hilden, Germany). In practice, 0.5 L of water was subjected to ultrafiltration to obtain a solid mass on a membrane filter (0.45 µm). DNA was then extracted from the collected filters, following the manufacturer’s instruction. The concentration and quality of the purified DNA was analysed on the NanoDrop Spectrophotometer (Thermo Fisher Scientific, Wilmington, USA). Five nanograms of purified DNA was used in PCR to prepare an amplicon library using the NEXTflex 16S V4 Amplicon-Seq Kit 2.0 (Bioo Scientific Corporation, Austin, TX, USA), following the provided protocol, which has previously been described in detail^21^. The applied specific 16S V4 forward primer (5′-GACGCTCTTCCGATCTTATGGTAATTGTGTGCCAGCMGCCGCGGTAA-3′) and reverse primer (5′-TGTGCTCTTCCGATCTAGTCAGTCAGCCGGACTACHVGGGTWTCTAAT-3′) were provided by the manufacturer and included in the sequencing kit. The library was prepared in triplicate for each sample. The final concentration of each library was measured on a Qubit Fluorometer (Life Technologies, Eugene, OR, USA) using the Quant-IT dsDNA HS Assay Kit (Invitrogen, Carlsbad, CA, USA). Pooling of all prepared libraries was achieved after concentration normalization using the SequalPrep Normalization Plate Kit (Thermo Fisher Scientific, Wilmington, USA). The pooled library was analysed on the Agilent 2100 Bioanalyzer system using the Agilent High Sensitivity DNA Kit (Agilent, CA, USA). It indicated a single band/unique product at 451 bp. The multiplexed library was sequenced on the Illumina MiSeq system using the MiSeq Reagent Kit V3, 600 Cycles (Illumina Inc., San Diego, CA, USA), following the default standard procedures.

### Sequence data processing

The output sequence datasets were analysed using Microbial Genomics Module 2.0, added onto the CLC Genomic Workbench 10.1.1 (CLC Bio, QIAGEN Company, Aarhus, Denmark, https://www.qiagenbioinformatics.com/products/clc-genomics-workbench). The processing workflow consisted of four key components: quality filtration, OTU clustering, and alpha and beta diversity measures. Adapter and primer sequences were trimmed. Unqualified reads were trashed when the quality score was less than 20 or a higher number of ambiguous nucleotides (more than two) were detected. The average length after trimming was between 220–230 bp. Chimeric sequences and singletons were detected and discarded. The remaining qualified reads were used to characterize OTUs based on a reference database (Greengenes v_13_5)^39^ at a 97% identity level. The bacterial alpha diversity of each sample was estimated in rarefaction analysis with a depth cutoff at 50,000 reads. The bacterial beta diversity applied the Euclidean distance criterion (EDC) to estimate the community similarities between the examined samples. All sequence data are available at NCBI Sequence Read Archive, under accession number SRR10835654-669, as part of BioProject PRJNA599104.

### Statistical analyses

Alpha diversity differences were tested using a two-tailed Student’s t-test at 0.05 significance. This was performed in the XLSTAT-ECOLOGY statistical software package version 2019.1.1 (Addinsoft 2020, Boston, USA, https://www.xlstat.com). A hierarchical clustering heat map was created to elucidate the bacterial community similarities/relatedness (pairwise) among all examined samples. It was conducted on a subset of top 700 OTUs, based on the EDC using the trimmed mean of M-values and Z-score (standard deviation numbers from the population mean) normalizations. This was further supported by the PERMANOVA analysis, performed to ascertain statistical significance of the clusters (p-value = 0.00001). These tests were executed using a package of functional features included in the Microbial Genomics Module (CLC Bio, QIAGEN Company, Aarhus, Denmark, https://www.qiagenbioinformatics.com/products/clc-genomics-workbench). Furthermore, the LEfSe tool^40^ was applied to identify the responsible bacterial members, accounting for community discrepancy between cold and warm seasons. The formatted abundance table of bacterial classes was uploaded to the Galaxy/Hutlab application web-based platform (Biostatistics Department, Harvard School of Public Health, Boston, MA, USA, https://huttenhower.sph.harvard.edu/galaxy) for pairwise comparison. Statistical significance of the comparison was determined by the Wilcoxon rank-sum test at the alpha value of 0.05. The identified features characterising the microbial differences among samples were processed using LDA, with a threshold score set at 2.0. Beyond that, RDA was carried out to determine the key abiotic environmental variables driving seasonal changes of the bacterial community based on the Pearson correlation test, with a statistical significance level higher than 95% (p < 0.05). It was performed using the most abundant bacterial phyla identified and determined physico-chemical parameters (abiotic environmental factors). Based on the computed scores of explanatory variables, only those with the uppermost weights and significant relations between the biotic and abiotic factors were included in the RDA plot. The RDA was performed in the XLSTAT-ECOLOGY statistical software package version 2019.1.1 (Addinsoft 2020, Boston, USA, https://www.xlstat.com).

### Artwork

The figure presenting the study site location (Fig. 1) is based on a satellite image and topographical map of Norway provided by the Norwegian Institute of Bioeconomy Research (NIBIO). The Institute’s geographical data is documented in the NIBIO’s primary map service the Source/Kilden (https://kilden.nibio.no). All maps and images collected in the Source/Kilden are open, and hence, they can freely be saved or printed (https://www.nibio.no/en/subjects/soil/national-land-resource-map?locationfilter=true).

Figures containing results and statistics (Figs. 2, 3, 4, 5) were generated using tools in the CLC Microbial Genomics Module version 2.5.1 (CLC Bio, QIAGEN Company, Aarhus, Denmark, https://www.qiagenbioinformatics.com/clc-microbial-genomics-module-latest-improvements), Galaxy version 1.0 (the Huttenhower Lab, Harvard School of Public Health, Boston, MA, USA, https://huttenhower.sph.harvard.edu/galaxy), and XLSTAT-ECOLOGY version 2019.1.1 (Addinsoft 2020, Boston, USA, https://www.xlstat.com).

## Supplementary information

Supplementary Data.

Supplementary Figure.

## Data Availability

The datasets generated during this study are available from the corresponding author upon reasonable request.
