# Supplementary figures and images for: Seasonal dynamics of lotic bacterial communities assessed by 16S rRNA gene amplicon deep sequencing
Source: Sci Rep. 2020 Oct 2;10:16399. doi: 10.1038/s41598-020-73293-9 (PMC7532223; doi:10.1038/s41598-020-73293-9)

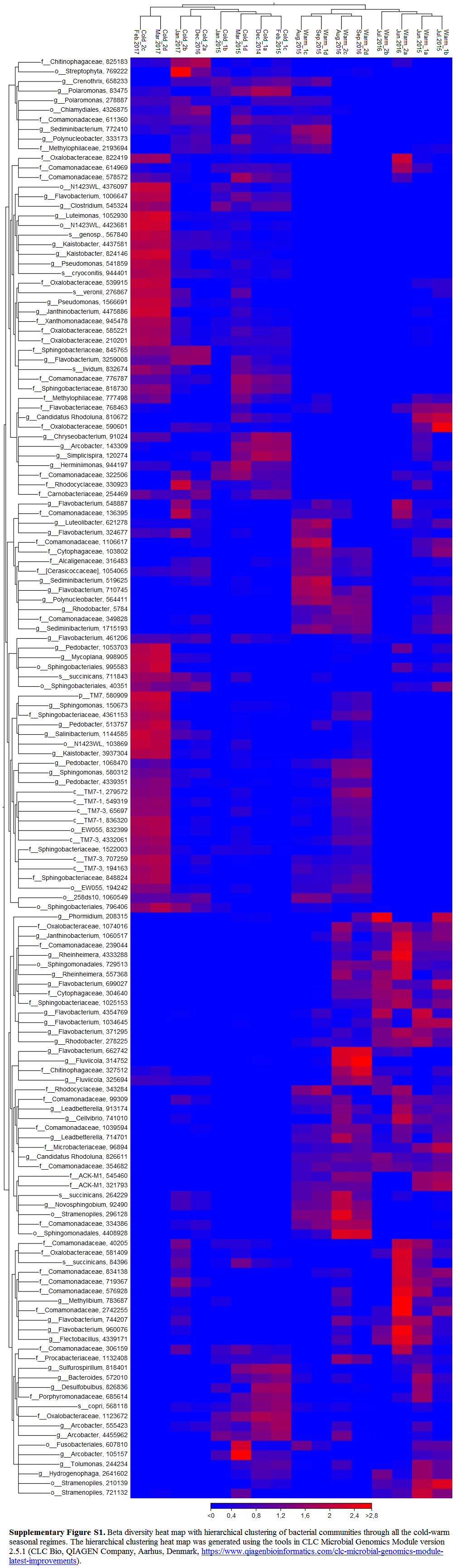

Supplement: Supplementary file 2 — Supplementary Figure. [file 41598_2020_73293_MOESM2_ESM.tif]
